# Ultrasound-Guided Radiofrequency Ablation and Pulsed Radiofrequency Treatment for Chronic Lameness Due to Distal Forelimb Disease in Horses: A Pilot Study

**DOI:** 10.3390/ani15162341

**Published:** 2025-08-10

**Authors:** Martina Amari, Federica Alessandra Brioschi, Luigi Auletta, Giuliano Ravasio

**Affiliations:** Department of Veterinary Medicine and Animal Sciences—DIVAS, Università degli Studi di Milano, 26900 Lodi, Italy; martina.amari@unimi.it (M.A.); federica.brioschi@unimi.it (F.A.B.); luigi.auletta@unimi.it (L.A.)

**Keywords:** chronic pain, thermal radiofrequency, interventional technique, neuromodulation, palmar digital nerve, Wallerian degeneration

## Abstract

Radiofrequency ablation (RFA) and pulsed radiofrequency (PRF) are non-pharmacological techniques employed in humans for chronic pain, but their veterinary application is unexplored. This pilot study evaluated clinical effects of RFA and PRF in twenty-four horses with chronic distal forelimb lameness. Ultrasound-guided RFA (N = 8; 60–90 °C, 2–8 min) or PRF (N = 16; 42 °C; 12 min) was applied to palmar digital nerves. Lameness was scored (American Association of Equine Practitioners scale) at baseline and monthly for six months (T1-T6). At T2, partial- and non-responders in both groups received PRF. Complications and return to previous work were recorded. At T2, the PRF group had significantly lower lameness scores (1, 0–3) than the RFA group (3, 2–4; *p* < 0.001) and significantly improved from baseline (3, 2–4; *p* < 0.01). RFA caused more complications (N = 6) than PRF (N = 1; *p* < 0.001), including increased lameness and allodynia. Sixteen horses (RFA: N = 7; PRF: N = 9) were retreated at T2. Overall, lameness significantly improved from T2 (2, 0–4) to T6 (0, 0–3; *p* < 0.001). At T6, 83% (19/23) of horses resumed previous work. RFA was ineffective and caused complications, whereas PRF appeared safer and more effective. Two PRF treatments yielded better outcomes with fewer side effects and may help manage lameness and associated pain for up to six months.

## Simple Summary

Chronic lameness is a common and painful condition in horses, often unresponsive to traditional treatments and significantly impairing quality of life. This study explored the use of radiofrequency, a non-pharmacologic, minimally invasive technique widely used in human medicine to treat chronic pain. Two radiofrequency modalities were tested: a heat-based approach called radiofrequency ablation, which produces long-lasting neurolysis by applying high temperatures, and a gentler method called pulsed radiofrequency, which alters the nerve function without causing damage. Horses with chronic forelimb lameness were treated using one of the two techniques. The pulsed method significantly reduced lameness in 81% of horses and caused very few side effects. In contrast, the heat-based method did not improve lameness and often led to complications, such as increased lameness and allodynia. Horses that did not improve after the first treatment often improved after receiving a second pulsed radiofrequency session. After six months, 83% of horses had returned to their previous level of work. These findings suggest that pulsed radiofrequency is a safe and effective option for managing chronic lameness in horses, offering a promising alternative to long-term medication or invasive surgery while improving the quality of life.

## 1. Introduction

Chronic distal forelimb lameness is a common, debilitating and painful condition in horses, which greatly affects quality of life [1]. It can be caused by a variety of distal limb pathologies, including navicular syndrome, deep digital flexor tendon tendinopathy, distal interphalangeal joint (DIPJ) osteoarthritis (OA), and collateral ligament desmopathy of the DIPJ [2,3,4]. In the pastern region, proximal interphalangeal joint (PIPJ) OA [5,6] and distal sesamoidean ligaments desmopathy [7,8] are common causes. When conservative approaches [9,10] fail to restore function or relieve pain [11], the impact on quality of life can be substantial, potentially leading to euthanasia [1]. Currently, palmar or plantar digital neurectomy is considered a last-resort option, though it carries risks such as neuroma formation [12,13] and increased risks of lower limb injuries [14]. However, no other considerable therapeutic advances have been made. A novel modality providing effective, long-lasting analgesia without adverse effects is therefore highly desirable.

Radiofrequency (RF) is a non-pharmacological, minimally invasive and low-risk technique widely used in human medicine for managing chronic pain unresponsive to conventional and rehabilitative treatments [15,16,17,18], including shoulder [15], knee [19,20], pelvic [21], facet joint [22,23], radicular [24,25], and neuropathic pain [26,27]. While in veterinary medicine RF has been applied in various contexts, including cardiac arrhythmia and tumor mass ablation [28,29], its perineural use for analgesic purposes has not yet been clinically described. The procedure involves inserting an insulated needle with a conductive tip close to the target nerve, which is connected to a high-frequency electric current generator that creates a small electric field at the tip and thus heat. Depending on the temperature reached, RF is classified as either radiofrequency ablation (RFA) or pulsed radiofrequency (PRF). RFA, operating at 60–90 °C, produces thermal neurolysis [30] via protein denaturation and coagulative necrosis [31]. Histopathological studies in rats [32,33] and human cadavers [34] showed that RFA induces immediate structural changes, such as collagen coagulation, edema, hyper-eosinophilia, and cytoplasmic vacuolization, also observed on palmar digital nerves (PDNs) of equine cadavers [35]. In rats and dogs, these changes are followed by Wallerian degeneration within 2–3 weeks, characterized by axonal swelling, fragmentation, and distal demyelination [36,37], which impairs nociceptive transmission and conduction [37] and contributes to delayed analgesia [36].

In contrast, PRF delivers a low-energy electrical field in rapid pulses, maintaining tissue temperature below 42 °C, thus avoiding irreversible coagulative necrosis [31]. Its analgesic action is primarily neuromodulatory [38,39], involving complex interactions with nociceptive signaling, ion channel regulation, synaptic function, and immune activity [38], with minimal histological impact on peripheral nerves, as demonstrated in experimental animal models [36,37,40]. Both RFA and PRF are intended for pain management rather than functional recovery. However, when neuropathic pain is suspected—common in treatment-resistant cases—the neuromodulatory effects of PRF may support functional improvement by influencing local neuronal activity [41].

The medial and lateral PDNs provide sensory innervation to the equine distal forelimb [42,43]. An abaxial sesamoid nerve block desensitizes the foot, middle phalanx, DIPJ, PIPJ, and associated soft tissues [44,45,46]. Therefore, RF targeting the PDN at this level may offer a promising therapeutic option in equine medicine. If proven to be effective, RF could be integrated into multimodal analgesic protocols as a minimally invasive alternative to surgery, potentially enhancing long-term management of chronic, treatment-resistant lameness. However, the use of RFA must be restricted to non-competing horses, given ethical and welfare concerns regarding sensory nerve desensitization in performance animals. In this context, RFA should only be considered when a return to athletic activity is no longer planned, as the primary goal is reducing lameness and associated pain and improving quality of life. A recent cadaveric study on the equine forelimb demonstrated the feasibility of ultrasound (US)-guided RF needle placement close to the PDN in the pastern and fetlock regions [35] and confirmed that RFA induced axonal thermal damage and coagulation proportional to treatment intensity. However, clinical studies are needed to evaluate efficacy and potential complications in vivo.

This pilot study aimed to evaluate the clinical outcomes of RFA and PRF in horses with chronic distal forelimb lameness, comparing their effectiveness, complications, and duration of lameness improvement. The hypothesis was that both RFA and PRF would be effective in reducing lameness and associated pain, with PRF offering lesser and shorter relief and RFA associated with a higher incidence of complications.

## 2. Materials and Methods

The present study complied with ethical standards, and it was conducted under the approval of the Institutional Ethical Committee for Animal Care of the University of Milano (OPBA_51_2023; 19/05/2023). Informed written consent for the proposed procedure and the use of data for scientific purposes was obtained by the owners of all horses enrolled in the study.

### 2.1. Animals

All horses referred to the Veterinary Teaching Hospital of the Department of Veterinary Medicine and Animal Science, University of Milano, with a history of chronic lameness were prospectively evaluated for enrollment in the study during the period May 2023–January 2025.

Inclusion criteria comprised horses with a history of at least six months of chronic mono- or bilateral distal forelimb lameness, graded 2 to 4 according to the American Association of Equine Practitioners (AAEP) lameness scale (0 = no perceptible lameness; 5 = non-weight-bearing lameness) [47], and a positive response to abaxial sesamoid nerve blocks (ASNBs) with 1.5 mL of 2% lidocaine (Lidocaina 2%; ECUPHAR ITALIA S.r.l, Milan, Italy) [44]. In cases of chronic bilateral lameness, both limbs must have a positive response to ASNBs, with the AAEP lameness score assigned based on the more severely affected limb. All evaluations were performed by three equine veterinarians with more than twenty years of experience in equine orthopedics; each veterinarian evaluated the same horse at all time points, blinded to the treatment group. Horses were observed turning by hand, moving away from and toward the examiner in a straight line at a walk and at a trot on a hard surface (asphalt/concrete/compacted sand). Then, they were lunged in a circle (diameter approximately 10 meters) for two minutes at a trot in each direction on a grass surface. All horses had to display alterations consistent with a chronic musculoskeletal disorder (e.g., navicular syndrome, OA of DIPJ) [48], confirmed by radiographic and/or magnetic resonance or computed tomography examinations. Horses were eligible for inclusion if lameness and activity impairment persisted despite systemic nonsteroidal anti-inflammatory drug (NSAID) administration or intra-articular corticosteroid injections, or if adverse effects were reported following their use. Exclusion criteria included horses weighing < 200 kg, aging ˂ two years, and displaying an American Society of Anesthesiologists physical status of ≥ IV based on clinical and laboratory findings. Additionally, horses with suspected or diagnosed acute or chronic laminitis, those failing to complete the six-month follow-up, or those receiving additional pharmacological or non-pharmacological treatments during the study period were excluded. For each horse enrolled in the study, data collection included age, body weight, breed, affected forelimb (right, left, or bilateral), and work type. Age and breed were recorded from the horse’s passport, body weight was measured using a large-animal scale, and work type—categorized as dressage, flatwork, jumping, paddock, trekking, or western—was reported by the owner. Furthermore, a definitive orthopedic diagnosis was recorded for each horse and subsequently classified into three pathological categories: osseous, joint, or tenodesmic disorders.

### 2.2. Study Design

This was a prospective, randomized controlled trial, single-blinded cohort pilot study evaluating the RFA and PRF treatments in horses with chronic lameness due to distal forelimb disease. Horses were randomly divided into two treatment groups by using an online tool (https://ctrandomization.cancer.gov/tool/; accessed on 30 May 2023), with a PRF to RFA ratio of 2:1. This allocation ratio was chosen considering the higher rate of complications for the RFA treatment reported in humans [16,49,50]. For the RFA group, according to the parameter settings evaluated in the cadaveric study [35], horses were randomly allocated to one of the four treatment settings: LOW treatment (60 °C, 6 min), MEDIUM treatment (70 °C, 4 min), HIGH treatment (90 °C, 2 min), and VERY HIGH treatment (80 °C, 8 min). For the PRF group, all horses received pulsed treatment at 42 °C for 12 min. In case of bilateral lameness, the same procedure with the same parameter settings was performed on both limbs. At the end of the procedure, a protective bandage was applied, and the horse was monitored in a box stall for two hours; discharge occurred only after confirming the absence of immediate post-sedation or post-RF complications, such as restlessness or pain at the treated site. All horses were rested for the first month after treatment, and firocoxib 0.1 mg/kg once daily orally (Equioxx pasta orale 8.2 mg/g; Audevard, Clichy, France) was administered during the 30 days following treatment. Horses underwent subjective lameness evaluation before treatment (T0) and every month after treatment for six months (T1 to T6), conducted by the same blinded equine veterinarian at the horse’s home facility. At each time, a lameness score from 0 to 5 was assigned using the AAEP lameness scale [47]. In case of bilateral lameness, the AAEP lameness score assigned based on the more severely affected limb. The treatment was repeated at T2 if lameness did not improve or if only partial improvement, i.e., one degree of lameness, was observed. Horses requiring a second treatment were transported back to the Veterinary Teaching Hospital for re-treatment. In both groups, the RF treatment was repeated with the PRF settings since, as expected, a higher number of complications were observed in the RFA group.

### 2.3. Radiofrequency Procedure

**Figure 1:**
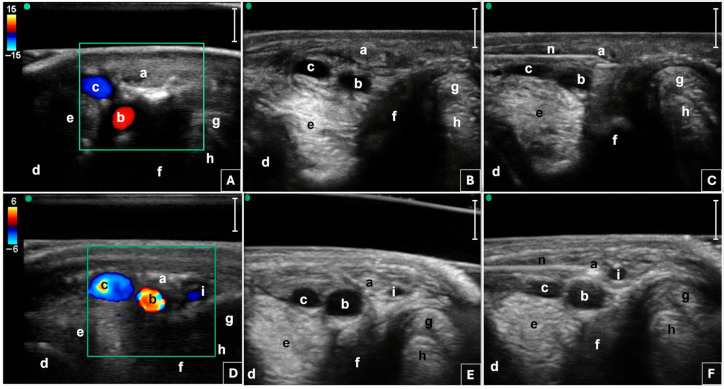
Transverse palmaro-lateral (**A**,**B**,**C**) and palmaro-medial (**D**,**E**,**F**) ultrasonographic images of the equine fetlock at the level of the proximal portion of the lateral and medial proximal sesamoid bones, respectively. Basal ultrasonographic scans (**B**,**E**). Use of color flow Doppler (**A**,**D**) to facilitate the identification of blood vessels and to distinguish them from the nerve. Ultrasound-guided in-plane positioning of the radiofrequency needle using the palmaro-lateral (**C**) and palmaro-medial (**F**) approaches, as described by Amari and colleagues (2024) [35]. Green dot, probe marker pointing dorsally; a, palmar digital nerve; b, palmar digital artery; c, palmar digital vein; d; third metacarpal bone; e, branch of the suspensory ligament; f, proximal sesamoid bone; g, superficial digital flexor tendon; h, deep digital flexor tendon; i, ergot vein; n, radiofrequency needle; bars equal to 5 mm. Note that the letters referring to even anatomical structures correspond to lateral structures in panels (**A**–**C**), and to medial structures in panels (**D**–**F**).

Prior to the procedure, food was withheld for eight hours. Each horse was sedated with intravenous acepromazine (0.03 mg/kg) (Prequillan 1%; FATRO S.p.A., Ozzano dell’Emilia, Italy), xylazine (0.5–1 mg/kg) (Nerfasin 100 mg/mL; P.H. Farmaceutici S.r.l., Milan, Italy), and butorphanol (0.02 mg/kg) (Nargesic 10 mg/mL; ACME S.r.l., Cavriago, Italy) and restrained in a standing position within a horse stock in a quiet, clean area. The fetlock region was clipped, aseptically prepared, and ASNBs [44] were performed with 3 mL of lidocaine 2% (Lidocaina 2%; ECUPHAR ITALIA S.r.l, Milan, Italy) each. A dispersive return pad electrode (GD-pad Corded; Diros Technology Inc., Markham, Ontario, Canada) was placed on the dorsal forearm and connected to an RF generator (OWL URF-3AP RF Generator; Diros Technology Inc., Markham, Ontario, Canada). An acoustic standoff pad (Standoff pad; Esaote, Genova, Italy) was placed between the skin and the probe to enhance visualization of superficial structures. To improve further acoustic coupling, a large amount of ultrasound gel (Ultrasound Gel; GIMA S.p.A., Gessate, Italy) was applied to the probe. Applying the technique previously described by Amari and colleagues (2024) [35], an anesthetist experienced in US-guided loco-regional anesthesia examined the fetlock region, using a portable US system (Sonosite M-Turbo; Sonosite Inc., Bothell, WA, USA) mounting a high-frequency 6–13 MHz linear array transducer (HLF38x; Sonosite Inc., Bothell, WA, USA), a scanning depth of 2.2–2.7 cm, and adjusting the frequency to obtain the clearest image possible. The US probe was positioned transversely to the metacarpophalangeal joint with the marker pointing dorsally, using a palmaro-lateral and a palmaro-medial approach, in order to identify the lateral and medial neurovascular bundles (Figure 1).

**Figure 2:**
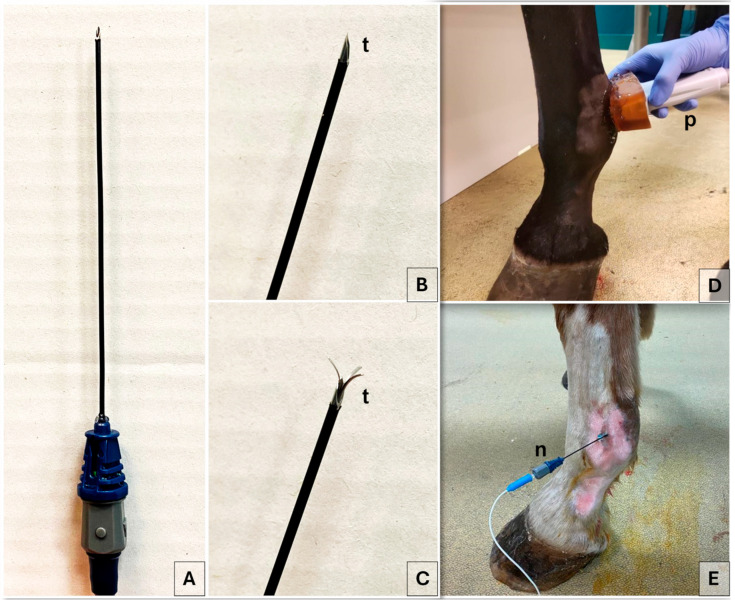
Radiofrequency needle employed in the present study (**A**). Image of the active tip closed (**B**) and with three tines opened (**C**). Ultrasound probe positioning on the medial aspect of the fetlock region (**D**). Radiofrequency needle positioning in the lateral aspect of the fetlock region (**E**). n, radiofrequency needle; p, ultrasound probe; t, active tip.

Specifically, the probe was positioned at the level of the proximal portion of the lateral and medial proximal sesamoid bones, respectively. Color flow Doppler was applied to facilitate the identification of blood vessels and to distinguish them from the nerve (Figure 1). The palmar digital artery (PDA) and palmar digital vein, as well as the ergot vein in the medial aspect, were superficial to the third metacarpal bone, the lateral or medial branch of the suspensory ligament and the corresponding proximal sesamoid bone [35]. The lateral and the medial PDNs were identified superficial to the lateral and medial PDAs, respectively, before branching into the lateral and medial proper PDNs and their corresponding dorsal PDN branch of the proximal phalanx [51]. An 18-gauge, three-tined, 5 mm active tip, 100 mm RF needle (RF Trident™ Cannulae; Diros Technology Inc., Markham, ON, Canada) (Figure 2) was inserted using an in-plane US technique and an approaching angle of 0 to 30° to the sagittal plane until the needle tip reached the target PDN, thus maintaining a 90° needle inclination relative to the nerve axis (Figure 1).

For the lateral PDN, the RF needle was advanced in a dorso-lateral to palmaro-medial direction, while for the medial PDN, it was introduced in a dorso-medial to palmaro-lateral direction (Figure 2). Based on previous cadaveric findings, the anesthetist consistently targeted a tip-to-nerve US distance of less than 2 mm [35]. Hence, RF needle tines were deployed under real time US-guidance, to visualize the needle tip close to the nerve and confirm correct positioning. Color flow Doppler was employed to prevent inadvertent contact between the needle tip and vascular structures. The treatment was performed first on the lateral side and then on the medial site.

### 2.4. Outcome Measure

The short-term outcome was assessed at two months post-treatment (corresponding to T2 for single treatments and T4 for double treatments), while the long-term outcome was evaluated at six months (corresponding to T6). Specifically, the RF treatment was deemed successful if lameness improved by at least two degrees within two months following the RF. A partial success was defined as an improvement of only one degree following the RF. Treatment failure was defined when no improvement was observed or when lameness worsened after the RF.

Adverse events were evaluated at each follow-up (T1–T6) by the same blinded equine veterinarian, and the treatment site was inspected for signs of swelling, heat, discharge, or altered sensitivity. Particularly, allodynia was clinically assessed by gently stroking the treated fetlock area with the fingertips. A marked pain response (e.g., withdrawal, flinching, or agitation) in the absence of visible local inflammation, signs of infection, or abnormalities on palpation of the neurovascular bundle was interpreted as indicative of peripheral sensory neuropathy consistent with allodynia. Neuroma formation was suspected during clinical examination if the following findings were observed: localized thickening along the course of the treated PDN, focal pain elicited by palpation at the treatment site, increased lameness, and a positive response to local diagnostic analgesia. Neuroma formation was considered clinically confirmed when ultrasonographic findings showed abnormally enlarged nerves of variable echogenicity. In addition to scheduled follow-ups, referring veterinarians and owners were instructed to monitor the horse’s clinical status and report any changes occurring between scheduled visits. Any complication arising from RF treatments, including hematoma formation, regional swelling, transient post-procedural discomfort, thermal burns, infection, neuropathic pain, and permanent nerve and/or tissue damage, was recorded. Worsening of the lameness after RF treatment was recorded as a complication, as well. The complications were classified according to the guidelines delineated by LeBlanc and colleagues (2021) for small animals [52], which were adapted for use in horses, categorizing them based on anatomical and/or pathophysiological factors and grading their severity on a scale from 1 to 5 (mild, moderate, severe, life-threatening, or fatal). Furthermore, their correlation with RF treatment was assessed using five likelihood categories: unrelated, unlikely, possible, probable, or definite [52]. At T6, horse owners were contacted to assess their horses’ return to work. Horses were classified as having “returned to their previous level of work” if they had resumed the same work as before the onset of lameness. Those who had retired from work entirely or were performing at a reduced level were categorized as “not returned to their previous level of work”.

### 2.5. Statistical Analysis

For a statistical purpose, the study was divided into two parts. All tests were two-tailed, if applicable, and significance was set at *p* < 0.05. Sample size was calculated a priori with G*Power© (v. 3.1, Franz Faul, Edgar Erdfelder, Albert-Georg Lang, and Axel Buchner, 2006, 2009, Kiel, Germany). For the first part of the study, a repeated measure analysis of variance with both within- and between-group comparisons was performed for two groups (RFA and PRF) at three time points (T0, T1, T2), assuming an effect size f(V) = 0.8, α = 0.05 and a power of 80%. For the second part of the study, a repeated measure analysis of variance with within-group comparisons only was used, as all horses in both groups were retreated with PRF across five time points (T2, T3, T4, T5, T6), with an effect size f = 0.2, a correlation among repeated measures of 0.7, ε = 1.0, α = 0.05 and a power of 80%. The first part of the study (from T0 to T2), involving the within- and between-group (RFA and PRF) comparisons, required a minimum total sample size of 18 horses, while the sample size analysis for the second part of the study, involving only within-group comparisons (PRF) and five time points (from T2 to T6), needed a minimum number of 20 horses.

Dedicated software for statistical analysis was used to perform all the evaluation described hereafter (JMP Pro, v. 17.0, SAS Institute, Cary, NC, USA; Prism 10.1.1, GraphPad Software, Inc., San Diego, CA, USA). Normality was tested with the Shapiro–Wilk W test, and data reported as mean ± standard deviation (SD) when parametric and as median (range) when non-parametric. The z test for proportions was applied to compare the complication rate between the two groups. A mixed effect model, analysis of variance for repeated measures with Geisser–Greenhouse correction, was applied to compare lameness degrees over time between the two treatment groups from T0 to T2. Post hoc, the two-stage step-up Benjamin, Krieger, Yekutieli method for multiple comparisons by controlling the false discovery rate was applied to identify between-treatment differences at each time point as well as between-time-point differences within treatment groups. A non-parametric Friedman repeated measures approach was applied from T2 to T6 without the effect of the treatment group. A nominal logistic model was applied to evaluate the influence on the treatment outcome, at T2 and T4, categorized as successful, partial or unsuccessful, of the following variables: treatment group, forelimb affected (right, left, bilateral), age, body weight, sex (male, gelding, female), and pathological category. Moreover, the treatment setting (LOW, MEDIUM, HIGH, VERY HIGH) within the RFA group and overall was tested for influencing the treatment outcome. Post hoc tests to further explore significant associations were used; in particular, the z-test was applied to compare the proportion of successful, partial or unsuccessful treatments between the RFA and PRF groups, and the analysis of variance to compare the body weight between the treatment outcome classes. A similar statistical approach was applied to evaluate the influence of the affected forelimb (right, left, and bilateral), age, and pathological category on the return to the same level of activity at T6.

## 3. Results

### 3.1. Animals

**Figure 3:**
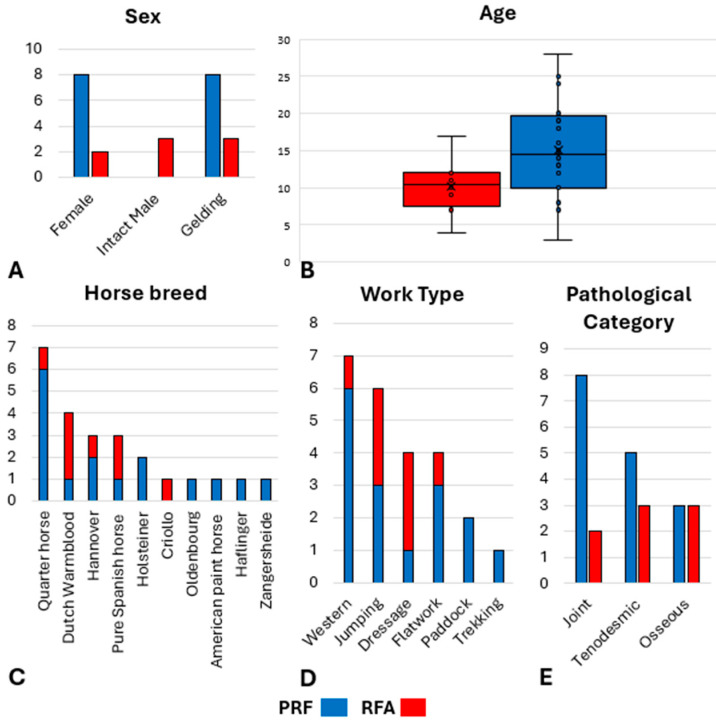
Signalment data for the horses included in the radiofrequency ablation group (RFA) and in the pulsed radiofrequency group (PRF). (**A**) Sex; (**B**) age (median, range); (**C**) number of horses by breed; (**D**) number of horses by work type; (**E**) number of horses by pathological category.

**Figure A1:**
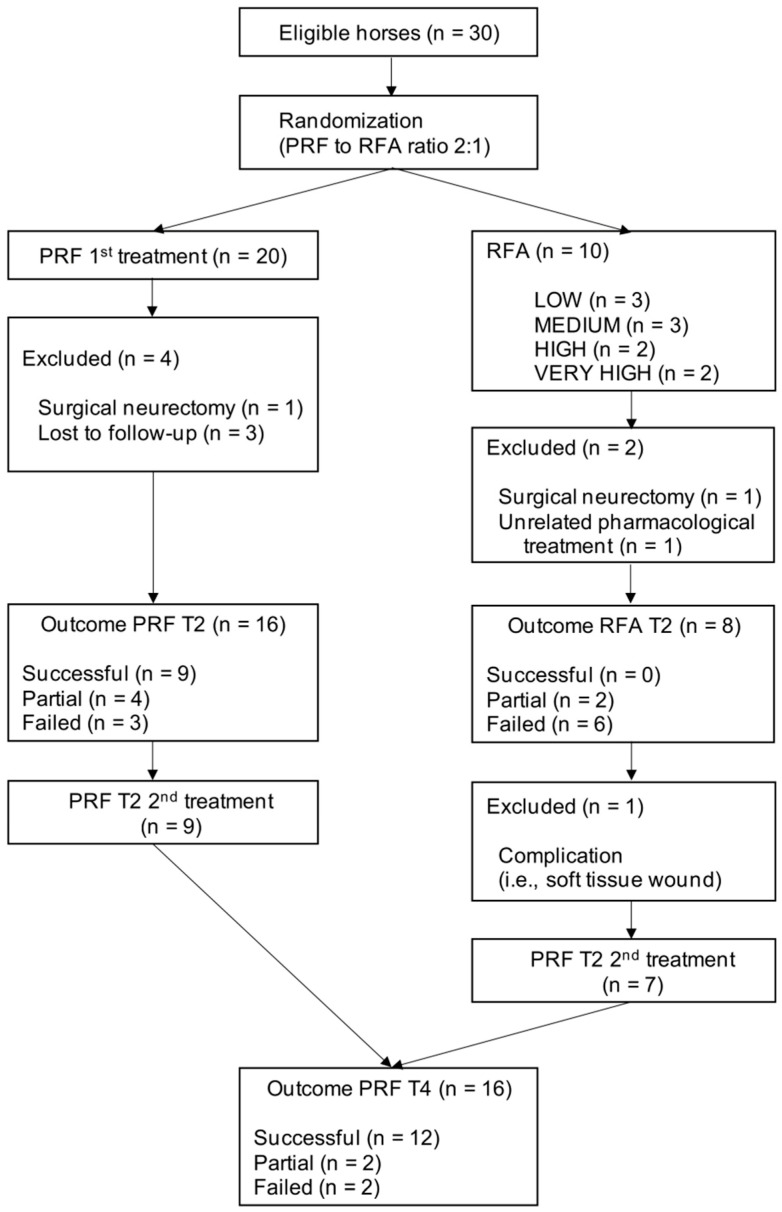
Patient flow chart. PRF: pulsed radiofrequency; RFA: radiofrequency ablation; T2: two months after the first radiofrequency treatment; T4: two months after the second radiofrequency treatment.

Thirty horses were initially recruited, but six of them were subsequently excluded (Figure A1); one horse (RFA group) was excluded due to unrelated pharmacological treatments administered during the follow-up period; two horses underwent surgical neurectomy of PDNs at T2 (one each from the PRF and RFA groups) at the request of the owners; and three horses (PRF group) were lost to follow-up after the first month. Consequently, 24 horses were finally enrolled in the study (RFA group—N = 8; LOW treatment, N = 3; MEDIUM treatment, N = 2; HIGH treatment, N = 1; VERY HIGH treatment, N = 2—and PRF group—N = 16) (Tables S1 and S2). Horses weighed 507 ± 63 kg and were 13 ± 6 years old. The signalment data for the horse breed, age, sex, work type and pathological categories represented in each group are reported in Figure 3.

On the overall sample, the median lameness score at T0 was 3 (2–4). Lameness scores assigned at T0 in each group, as well as the distribution of forelimbs affected by lameness, are shown in Table 1.

### 3.2. Outcome Measure

At T2, in the RFA group, the treatment was considered successful in zero, partial in two (one each from LOW and MEDIUM treatments), and failed in six (one each from HIGH and MEDIUM treatments and two each from VERY HIGH and LOW treatments) horses. In the PRF group, the treatment was considered successful in nine, partially successful in four, and failed in three horses. One out of 16 horses from the PRF group and six out of eight horses from the RFA group developed complications after the RF treatment; the rate of complications was significantly higher in the RFA group (*p* < 0.001). Particularly, one horse in the RFA (LOW treatment) group showed grade 1 edema localized at the treatment site following bandage removal. The swelling resolved within two days without no additional treatment beyond the NSAIDs required by the study and was classified as “definite” related to the RF treatment [52]. Two horses, one in the PRF group and one in the RFA group (HIGH treatment), developed increased lameness with moderate pain (grade 2) one week after treatment, and it was “probably” related to the procedure, according to LeBlanc and colleagues (2021) [52]. Lameness in the PRF horse increased from AAEP grade 2 to 3 at T1 and improved to grade 1 at T2, while the RFA horse worsened from AAEP grade 2 to 4 at T1 and improved to grade 3 at T2. As per the study protocol, both received analgesic therapy with firocoxib and underwent a second treatment with PRF. Three horses in the RFA group (VERY HIGH and LOW treatment) experienced bilateral allodynia on the treated PDNs, which occurred three to four weeks after the RFA and resolved within one month after the second treatment with the PRF settings. This peripheral sensory neuropathy was classified as mild (grade 1) and “definite” related to the RFA treatment [52]. Finally, a soft tissue wound in the lateral dorsal pastern region during paddock resting was recorded at T2 in one horse in RFA group (MEDIUM treatment). According to LeBlanc and colleagues (2021), the authors consider this event “possibly” related to the procedure (category 3) [52]. The complication, graded as severe (grade 3), required hospitalization and medical treatment and significantly impaired daily activities. A diagnosis of septic arthritis of the proximal interphalangeal joint was made. Consequently, the horse was excluded from further follow-up in the study. No neuroma formation was observed at any temperature or duration setting.

**Figure 4:**
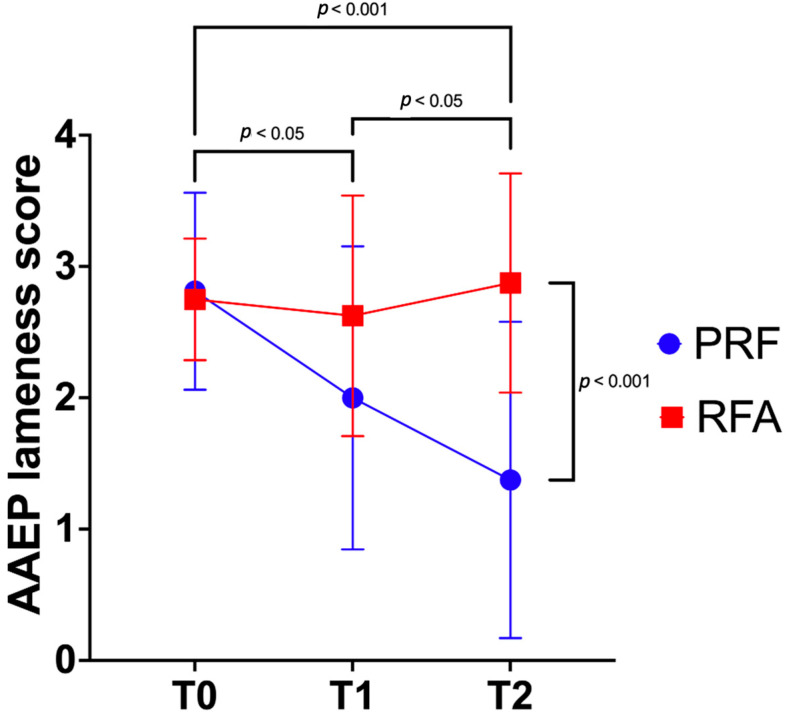
Mean and standard deviation of American Association of Equine Practitioners (AAEP) [47] lameness score in PRF (N = 16) and RFA (N = 8) groups at the baseline (T0), at one month (T1) and at two months (T2) after the pulsed radiofrequency and the radiofrequency ablation, respectively. Values expressed as median (range) are available in Table 2.

The lameness degree from T0 to T2 was significantly affected by the treatment group (*p* = 0.02), by the time (*p* = 0.04), and by their interaction (*p* = 0.03). Indeed, the PRF group showed a significantly lower AAEP lameness score compared to the RFA group at T2 (*p* < 0.001; Figure 4), while no significant differences were detected at T0 (*p* > 0.9) nor at T1 (*p* = 0.1).

Within the RFA group, no differences could be detected between the three time points. On the other hand, in the PRF group, the AAEP lameness scores decreased gradually and significantly over time. The AAEP lameness score in the two groups at each time point and within groups significant differences are summarized in Table 2.

At T2, the treatment outcome was significantly influenced by the treatment group (*p* = 0.003). Indeed, none of the horses in the RFA group were successfully treated, and a significantly higher proportion of horses from the RFA group were classified as unsuccessfully treated compared to the PRF group (*p* = 0.01). No influence could be detected by the affected forelimb (*p* = 0.2), age (*p* = 0.7), sex (*p* = 0.06), body weight (*p* = 0.2), and pathological category (*p* > 0.9). The treatment settings did not influence the treatment outcome within the RFA group (*p* = 0.5) nor overall (*p* = 0.08).

At T2, all partially or unsuccessfully treated horses (N = 14) underwent a second treatment with PRF parameter settings. Moreover, two horses in the PRF group that were deemed successful at T2—from grades 3 and 4 at T0 to grades 1 and 2, respectively—were re-admitted for the second treatment to further reduce the degree of lameness at the request of the owners. Hence, at T4, treatment was deemed successful in 12, partial in two, and unsuccessful in two horses. At T4, no complications were recorded in any horse after the PRF treatment.

**Figure 5:**
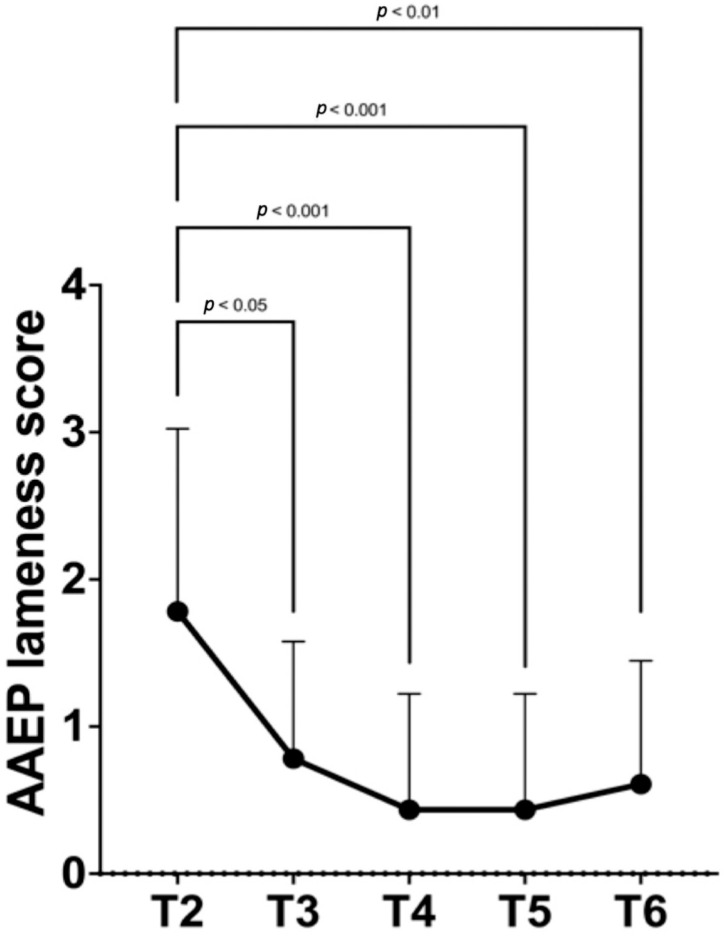
Mean and standard deviation of American Association of Equine Practitioners (AAEP) [47] lameness score in horses (N = 23) from second (T2) to sixth month (T6) after the pulsed radiofrequency (PRF) or the radiofrequency ablation (RFA) treatment at the baseline. At T2, sixteen horses underwent a second treatment with PRF because of a partial/failed response to the first treatment or to further reduce the degree of lameness. Values expressed as median (range) are available in Table 3.

The AAEP lameness score from T2 to T6 had significantly reduced (*p* < 0.001; Figure 5).

The AAEP lameness score at each time point and between time points significant differences are summarized in Table 3.

At T4, the treatment outcome was not influenced by the forelimb affected (*p* = 0.3), age (*p* = 0.2), body weight (*p* = 0.2), sex (*p* = 0.3), and pathological categories (*p* = 0.1). At T6, 19/23 (83%) horses returned to the previous level of work, while 4/23 (17%) horses did not return to the previous level of work. The return to the previous level of work at T6 was not affected by the affected forelimb (*p* = 0.6), age (*p* = 0.9), and pathological category (*p* = 0.6).

## 4. Discussion

This study represents the first clinical investigation into the short- and long-term outcomes of RFA and PRF in horses with chronic distal forelimb diseases.

### 4.1. RFA Clinical Outcomes and Neuroablation

Contrary to the hypothesis, the RFA technique applied on PDNs did not improve lameness at one and two months post-treatment. This result contrasts with evidence from human literature, where several studies have demonstrated that RFA is effective [18,20] and, in many cases, more effective than PRF in alleviating pain [23,53,54]. Moreover, maximal pain relief in human patients is typically achieved between one month and three months after treatment [55]. As reported previously in humans [56] and dogs [57], in the present study, the lack of efficacy of RFA may be attributed to the high incidence of complications, which could have masked potential therapeutic effects. Notably, no significant differences were observed among treatment settings. Previous ex vivo studies have shown that lesion size increases with both temperature and treatment duration [58], with higher treatment intensities leading to more frequent nerve coagulation in equine forelimbs [35]. Therefore, better outcomes were expected with VERY HIGH and HIGH settings. However, the only two cases showing partial clinical improvement occurred with the MEDIUM and LOW settings, which may have minimized side effects. In contrast, more invasive settings may be associated with a higher incidence of complications, as reported in humans [32]. Nevertheless, these findings should be interpreted with caution given the limited sample size in the RFA subgroups.

In this clinical study, the ASNBs with 3 mL of lidocaine 2% were performed before RF needle insertion, in accordance with standard clinical practice to minimize procedural pain [59,60]. Provenzano et al. (2010) demonstrated that fluid pre-injection increased lesion size, probably by enhancing electric conductivity [61]. However, in this study, lesion size was not directly measured, and therefore any inference regarding lesion size remains speculative. Although larger lesions could threaten adjacent structures like the PDA [35], no tissue or vascular damage was recorded, likely due to the in vivo heat sink effect mitigating damage, as previously reported [62]. Nevertheless, future studies including imaging or histological assessment are warranted to correlate lesion size with clinical outcomes and potential complications.

These findings highlight the importance of balancing efficacy and safety, suggesting that excessively aggressive ablation may be counterproductive. Based on current results, RFA with the tested settings is not recommended as a first-line treatment. Further studies with larger and more homogeneous groups are needed to refine RFA parameters, particularly by exploring lower temperature settings that may enhance efficacy while minimizing the risk of adverse effects.

### 4.2. PRF Clinical Outcomes and Neuromodulation

Consistent with the initial hypothesis, the PRF technique demonstrated to be a safe and effective treatment for the chronic distal limb lameness in horses, representing a less invasive alternative to RFA. A single PRF application led to a progressive and significant lameness reduction, with scores significantly lower than the RFA group two months post-treatment. Similar trends of progressive pain relief were reported in human studies, with effects becoming significant within one to three months [25,63,64]. This delayed therapeutic effect may stem from PRF-induced plastic changes in pain transmission pathways, gradually leading to neuromodulation [65]. Indeed, electron microscopy studies on rats revealed structural alterations in peripheral nerves, including mitochondrial damage and disruption/disorganization of microfilaments and microtubules [40]. Interestingly, these effects appear to be more pronounced on unmyelinated C-fibers and myelinated A-δ fibers—both primarily involved in nociceptive transmission—than on thick A-β fibers, which are associated with non-nociceptive tactile sensations [40,66]. This selectivity may explain PRF’s ability to relieve pain while preserving some sensory function [40,66]. In the equine fetlock region, the PDN consists of myelinated sensory fibers, unmyelinated peptidergic, and unmyelinated sympathetic sensory fibers [42] with an approximate unmyelinated-to-myelinated fiber ratio of 4:1 [67]. Since most unmyelinated fibers transmit pain signals [67], PRF may be particularly suited for managing chronic pain in the equine distal limb (e.g., laminitis).

### 4.3. Complications: RFA vs. PRF

A significantly higher number of complications were recorded in the RFA group compared to the PRF group, supporting the study hypothesis. In human medicine, RF is generally considered low risk [49,68], with the most side effects being mild, transient, and self-limiting. The most common complication is transient post-procedural pain, often due to neuritis or neurogenic inflammation [17,68]. Other potential complications include bleeding, infection, localized swelling, and inadvertent nerve damage related to needle insertion [17]. RFA also carries risks of thermal burns [69] and neuropathic pain [70], which are not typically associated with PRF [41,68]. Moreover, RFA can only be applied to sensory nerves due to its risk of motor deficit [36], while PRF, producing minimal histological effects, is often used for nerves with motor fibers [16]. In the present study, similar procedure-related complications were observed. One horse developed localized swelling, while two experienced a transient increase in lameness, temporarily exceeding baseline values the first month. Given the anticipated inflammation and discomfort, all horses received post-procedural anti-inflammatory therapy. Hence, this preventive approach may have mitigated the occurrence of additional minor side effects. Mild allodynia was recorded in three RFA-treated horses, though of limited clinical significance. In humans, neuropathic pain is the most notable complication of RFA [49], manifesting as paresthesia, dysesthesia [71], allodynia [72], hyperalgesia, or deafferentation pain [73]. Two of the three cases of allodynia occurred in horses treated with the VERY HIGH RFA setting, consistent with the known association between lesion size and neuropathic risk [74]. Interestingly, human studies reported that such neuropathic complications resolved within weeks to months and were not debilitating [56,74,75,76]. However, it remains unclear whether allodynia in this study would have resolved naturally, as all three horses underwent PRF at T2. Indeed, PRF has shown efficacy in treating experimentally induced allodynia in rats [77,78], Further investigation is warranted to better elucidate this aspect.

### 4.4. Potential Risks Related to RF

RFA is designed to induce temporary denervation, as axonal regeneration generally occurs along the preserved architecture of the nerve [30,79]. For this reason, neuroma formation following RFA is rare in human medicine [80]. However, after aggressive treatments, the risk cannot be entirely excluded. Indeed, although the preserved epineurium typically lowers neuroma risk [79], severe axonal injury [50] or repeated ablations [81] may prolong pain relief due to delayed or reduced axonal regeneration, while simultaneously increasing the risk of neuroma-in-continuity formation [50], particularly if the perineurium is compromised [30]. By contrast, surgical neurectomy involves neurotmesis to achieve permanent denervation, which eliminates the structural scaffolding normally provided by the perineurium and epineurium [79]. This absence significantly increases the likelihood of disorganized axonal regrowth and, consequently, the formation of painful neuromas [79], with incidence rates ranging from 4% to 25% [13,82,83,84,85]. Furthermore, although only one human case report described trophic complications after RFA [86], neuroablative techniques could impair tissue trophism in horses, similarly to what has been observed after surgical neurectomy [87], particularly in cases involving permanent nerve damage. This risk could be minimal or potentially absent with PRF, due to its neuromodulatory mechanism, which is presumed to preserve or even modulate the nerve’s trophic function. Nonetheless, this aspect warrants further investigation. Lastly, typical complications of equine surgical neurectomy, such as deep digital flexor tendon rupture, DIPJ luxation, and subsolar injuries [88] due to complete distal limb desensitization, were not observed in the RFA group. However, these complications remain theoretically possible and could potentially occur beyond the six-month follow-up period of this study. Indeed, the etiology of the soft tissue wound in one RFA-treated horse remains uncertain, though it may be procedure-related. It may have resulted from a wound penetrating the PIPJ while in the paddock, due to sensory deficit from the treatment [69], or from a self-inflicted injury linked to neuropathic pain. Indeed, as previously discussed, this complication is often reported in human literature, and numbness, tingling and pricking are characteristic of this condition [89]. Similar discomfort due to peripheral neuropathy is a recognized cause of self-injury in horses, as it can trigger attempts to relieve distressing sensations [90]. Therefore, in this case, septic arthritis may have developed secondary to a self-inflicted injury of the pastern, which subsequently extended to involve the underlying joint.

### 4.5. PRF Second Treatment Strategy

Although PRF offers several advantages, its efficacy in human medicine is generally lower than RFA in both pain relief and duration [23,53]. Similarly, in this study, seven out of 16 horses in the PRF group experienced only partial or no improvement after a single treatment, supporting the initial hypothesis of a limited therapeutic effect in equine patients. Currently, standardized PRF parameters are lacking in humans, but increasing voltage [64,65], extending treatment duration, or repeating treatments (up to two or three times) [91,92] were proposed to enhance outcomes. In this study, to enhance the neuromodulation in the PRF group and given the higher expected complication rate with RFA, a second PRF treatment was administered in both groups. This choice was also supported by evidence suggesting PRF may be preferable in case of nerve damage, as could occur post-RFA [30,92]. Some studies also showed that combining RFA and PRF reduced post-treatment complications, but the PRF was applied immediately after RFA [93,94]. In the present study, 12 out of 16 horses receiving a second PRF treatment at T2 exhibited at least two degrees of lameness reduction by T4, suggesting two consecutive PRF applications may enhance neuromodulation. Additionally, combining RFA and PRF may have improved efficacy by targeting different biological pathways while mitigating RFA-related side effects. Nevertheless, given the complication rate observed with RFA, the authors consider repeated PRF treatments the preferable first-line strategy, while the sequential RFA-PRF treatment shows potential but requires a safer RFA setting and further study before endorsement. Lameness significantly decreased from T2 to T6, and 83% (19/23) of horses had returned to their previous level of work by this time. The four horses that did not were all in the PRF group: two showed partial and no response to the treatments, respectively; the other two initially responded positively, being sound for two and three months, respectively, but relapsed with one degree of lameness at T6, leading their owners to opt for rest. This outcome aligns with human studies, where pain relief duration varies widely from three to 24 months, depending on treatment site, technique, and individual response [16,18]. Given that horses with chronic lameness and associated pain may require lifelong medical management or may not respond adequately to conventional treatments, the PRF treatment appeared to be effective in partially or completely reducing chronic lameness in a high percentage of cases up to six months. Future studies in the equine patients may clarify the long-term efficacy beyond this follow-up.

### 4.6. Limitations

The main limitation of this study was the sole use of a subjective lameness assessment, unaccompanied by any objective evaluation method, alongside the inclusion of horses affected by bilateral lameness. Although the AAEP scoring was consistently applied by experienced blinded equine veterinarians and each horse was assessed by the same clinician to minimize variability, the lack of objective data limits precision and reproducibility. Moreover, this scale primarily measures lameness degrees and not necessarily pain intensity. Future studies should incorporate quantitative motion analysis to allow for a more accurate evaluation of bilateral lameness. Another limitation was the inability to determine whether outcomes were influenced by the RF procedure itself (i.e., the feasibility of the technique) or by the specific treatment modality applied (RFA and PRF). A previous equine cadaveric study reported a 77.5% success rate in needle placement close to the PDN, with higher failure in the fetlock than the pastern, likely due to greater nerve mobility and reduced skin-probe contact [35]. In the present clinical study, only PDNs in the fetlock region were targeted, potentially increasing failure risk. However, in vivo US equine nerve identification is reportedly more successful than in cadavers, likely aided by better echogenicity, blood flow, and color flow Doppler [95]. In this study, PDNs were consistently well visualized, and the use of anatomical landmarks and Doppler likely improved accurate US-guided needle placement. Still, minor limb movements during the procedure may have affected needle placement during treatment, making it unclear whether partial or negative outcomes were due to technical error or treatment inefficacy. A further relevant limitation was the absence of sham and control groups, which would have helped rule out confounding factors such as procedure-related complications and the effect of NSAID administration or rest. Although the outcome was assessed one month after firocoxib discontinuation, the potential residual pharmacological effects could not be excluded. Future studies in horses should optimize PRF protocols (e.g., longer application time) and refine RFA parameters by lowering temperature and/or exposure time to reduce complications. Long-term evaluation of single versus repeated RF treatments is also recommended.

## 5. Conclusions

In conclusion, this pilot study represented the first clinical investigation into the application of RF in veterinary medicine. Although these preliminary results should be interpreted with caution due to the small sample size and potential confounding factors, this study provides a foundation for future research aimed at validating and refining RF protocols for horses with chronic lameness and associated pain.

RFA failed to improve lameness and was associated with a significantly higher complication rate compared to PRF. Therefore, the RFA settings used in this study are not currently recommended. In contrast, PRF proved to be a safe and effective treatment, leading to significant and progressive lameness improvement over two months. A second PRF treatment resulted in sustained lameness reduction over six months, suggesting that two repeated PRFs may contribute to long-lasting improvement in lameness and quality of life. Overall, PRF seems to be a promising, minimally invasive option for managing chronic distal forelimb diseases in horses, potentially reducing the request for pharmacological therapy or surgical neurectomy. Integrating RF techniques with other pain management strategies could further improve outcomes through a multimodal approach to chronic lameness in horses.

## Figures and Tables

**Table 1:** Distribution (absolute counts and percentage of the total) of the enrolled horses according to treatment group (pulsed radiofrequency, PRF, and radiofrequency ablation, RFA), and distribution according to lameness degree and affected forelimbs at baseline (T0) in the overall sample and within the treatment groups.

Sample		Lameness Degree			Affected Forelimbs		
Group	Number of Horses	AAEP Score 2	AAEP Score 3	AAEP Score 4	Right	Left	Bilateral
**PRF**	16	6	7	3	5	3	8
**RFA**	8	2	6	0	4	3	1
**Total**	24	8 (33%)	13 (54%)	3 (13%)	9 (37.5%)	6 (25%)	9 (37.5%)

Lameness degree was assessed according to the American Association of Equine Practitioners (AAEP) lameness scale [47].

**Table 2:** Median (range) of AAEP lameness score in PRF (N = 16) and RFA (N = 8) groups at the baseline (T0), at one month (T1) and at two months (T2) after the pulsed radiofrequency and the radiofrequency ablation, respectively.

Group	T0	T1	T2	*Within Groups Mean Diff. (95% CI)*
**PRF**	3 (2–4) ^a,A^	2 (0–3) ^b^	1 (0–3) *^,c,B^	T1–T0 −0.8 (−1.5–−0.1) T2–T1 −0.6 (−1.5–−0.2)
**RFA**	3 (2–3)	3 (1–4)	3 (2–4) *	T0–T1 −0.1 (−0.9–0.6) T1–T2 0.2 (−0.7–1.2)
*Between groups mean diff. (95% CI)*	0.1 (−0.5–0.7)	0.6 (−0.3–1.6)	1.5 (0.5–2.5)	

Between group differences * for *p* < 0.001. Within-group differences are a > b and b > c for *p* < 0.05 and A > B for *p* < 0.01. Lameness degree was assessed according to the American Association of Equine Practitioners (AAEP) lameness scale [47]. diff.: difference; 95% CI: 95% confidence interval.

**Table 3:** Median (range) of AAEP lameness score in horses (N = 23) from second (T2) to sixth month (T6) after the pulsed radiofrequency (PRF) or the radiofrequency ablation (RFA) treatment at the baseline. At T2, sixteen horses underwent a second treatment with PRF because of a partial/failed response to the first treatment or to further reduce the degree of lameness.

T2	T3	T4	T5	T6
2 (0–4) ^a,A^	1 (0–3) ^b^	0 (0–3) ^B^	0 (0–3) ^B^	0 (0–3) ^B^
*Between groups mean diff. (95% CI)*	T3–T2 −1.0 (−1.6–−0.4)	T4–T2 −1.35 (−2.0–−0.7)	T5–T2 −1.3 (−2.0–−0.7)	T6–T2 −1.2 (−1.8–−0.5)

Within-group differences are a > b for *p* < 0.05 and A > B for *p* < 0.01. Lameness degree was assessed according to the American Association of Equine Practitioners (AAEP) lameness scale [47]. diff.: difference; 95% CI: 95% confidence interval.

## Data Availability

The original contributions presented in this study are included in the article/Supplementary Material. Further inquiries can be directed to the corresponding author(s).

## Supplementary Materials

The following supporting information can be downloaded at https://www.mdpi.com/article/10.3390/ani15162341/s1, Table S1. Signalment data for the horses included in the radiofrequency ablation group and in the pulsed radiofrequency group, including specific temperature and minute settings received, treated limb(s), breed, sex, weight, age, work type, pathological category, and specific pathology; Table S2: Summary of clinical outcomes and lameness scores per horse over the entire follow-up period. Individual horse data including treatment group, specific temperature and minute setting received, outcome after the first and (if applicable) second treatment, complications, return to previous work activity, and lameness scores at baseline and at each follow-up.

## Abbreviations

The following abbreviations are used in this manuscript:

AAEP	American Association of Equine Practitioners
ASNB	Abaxial Sesamoid Nerve Block
ASNBs	Abaxial Sesamoid Nerve Blocks
CI	Confidence Interval
CT	Computed Tomography
DIPJ	Distal Interphalangeal Joint
MRI	Magnetic Resonance Imaging
NSAID	Nonsteroidal Anti-inflammatory Drug
OA	Osteoarthritis
PDA	Palmar Digital Artery
PDN	Palmar Digital Nerve
PDNs	Palmar Digital Nerves
PIPJ	Proximal Interphalangeal Joint
PRF	Pulsed Radiofrequency
RF	Radiofrequency
RFA	Radiofrequency Ablation
SD	Standard Deviation
US	Ultrasound

## References

[1] Pollard D., Wylie C.E., Newton J.R., Verheyen K.L.P. (2020). Factors Associated with Euthanasia in Horses and Ponies Enrolled in a Laminitis Cohort Study in Great Britain. Prev. Vet. Med..

[2] Gutierrez-Nibeyro S., Werpy N., White N. (2012). Standing Low-Field Magnetic Resonance Imaging in Horses with Chronic Foot Pain. Aust. Vet. J..

[3] Koch D.W., Barrett M.F., Jackman B.R., MacDonald D., Goodrich L.R. (2020). Comparison of Lameness Outcomes in Horses with Acute or Chronic Digital Lameness That Underwent Magnetic Resonance Imaging. New Zealand Veter J..

[4] Pauwels F., Hartmann A., Alawneh J., Wightman P., Saunders J. (2021). Contrast Enhanced Computed Tomography Findings in 105 Horse Distal Extremities. J. Equine Vet. Sci..

[5] Himani H., Kumar A., Anand A., Singh N., Uppal V., Mohindroo J. (2019). Clinical Occurrence and Radiographic Diagnosis of Distal Limb Lameness in Equine. Indian J. Anim. Sci..

[6] Schaer T.P., Bramlage L.R., Embertson R.M., Hance S. (2001). Proximal Interphalangeal Arthrodesis in 22 Horses. Equine Vet. J..

[7] Sampson S.N., Schneider R.K., Tucker R.L., Gavin P.R., Zubrod C.J., Ho C.P. (2007). Magnetic Resonance Imaging Features of Oblique and Straight Distal Sesamoidean Desmitis in 27 Horses. Vet. Radiol. Ultrasound.

[8] Hawkins A., O’Leary L., Bolt D., Fiske-Jackson A., Berner D., Smith R. (2022). Retrospective Analysis of Oblique and Straight Distal Sesamoidean Ligament Desmitis in 52 Horses. Equine Vet. J..

[9] Van Weeren P.R., Back W. (2016). Musculoskeletal Disease in Aged Horses and Its Management. Vet. Clin. N. Am. Equine Pract..

[10] Daglish J., Mama K.R. (2016). Pain: Its Diagnosis and Management in the Rehabilitation of Horses. Vet. Clin. N. Am. Equine Pract..

[11] Flood J., Stewart A.J. (2022). Non-Steroidal Anti-Inflammatory Drugs and Associated Toxicities in Horses. Animals.

[12] Matthews B.G., Hurn S.E., Harding M.P., Henry R.A., Ware R.S. (2019). The Effectiveness of Non-Surgical Interventions for Common Plantar Digital Compressive Neuropathy (Morton’s Neuroma): A Systematic Review and Meta-Analysis. J. Foot Ankle Res..

[13] Gutierrez-Nibeyro S.D., Werpy N.M., White N.A., Mitchell M.A., Edwards R.B., Mitchell R.D., Gold S.J., Allen A.K. (2015). Outcome of Palmar/Plantar Digital Neurectomy in Horses with Foot Pain Evaluated with Magnetic Resonance Imaging: 50 Cases (2005–2011). Equine Vet. J..

[14] Madison J.B., Dyson S.J., Ross M.W., Dyson S.J. (2003). Treatment and Prognosis of Horses with Navicular Disease. Diagnosis and Management of Lameness in the Horse.

[15] Albishi W., Abudujain N.M., Bin Dakhil A., Alzeer M. (2023). The Utilization of Radiofrequency Techniques for Upper Extremity Pain Management. Pain Physician.

[16] Vanneste T., Van Lantschoot A., Van Boxem K., Van Zundert J. (2017). Pulsed Radiofrequency in Chronic Pain. Curr. Opin. Anaesthesiol..

[17] Senthelal S., Dydyk A.M., Mesfin F.B., StatPearls Publishing (2024). Ablative Nerve Block. StatPearls.

[18] Michaud K., Cooper P., Abd-Elsayed A., Kohan L. (2021). Review of Radiofrequency Ablation for Peripheral Nerves. Curr. Pain Headache Rep..

[19] Iannaccone F., Dixon S., Kaufman A. (2017). A Review of Long-Term Pain Relief after Genicular Nerve Radiofrequency Ablation in Chronic Knee Osteoarthritis. Pain Physician.

[20] Koshi E., Meiling J.B., Conger A.M., McCormick Z.L., Burnham T.R. (2022). Long-Term Clinical Outcomes of Genicular Nerve Radiofrequency Ablation for Chronic Knee Pain Using a Three-Tined Electrode for Expanded Nerve Capture. Interv. Pain Med..

[21] Seale C., Connolly B.R., Hulk K., Yu G.G., Nagpal A.S. (2021). The Use of Radiofrequency in the Treatment of Pelvic Pain. Phys. Med. Rehabil. Clin. N. Am..

[22] Russo M.A., Santarelli D.M. (2021). Development and Description of a New Multifidus-Sparing Radiofrequency Neurotomy Technique for Facet Joint Pain. Pain Pract..

[23] Tekin I., Mirzai H., Ok G., Erbuyun K., Vatansever D. (2007). A Comparison of Conventional and Pulsed Radiofrequency Denervation in the Treatment of Chronic Facet Joint Pain. Clin. J. Pain.

[24] Choi G., Ahn S.-H., Cho Y.-W., Lee D.-G. (2012). Long-Term Effect of Pulsed Radiofrequency on Chronic Cervical Radicular Pain Refractory to Repeated Transforaminal Epidural Steroid Injections. Pain Med..

[25] Van Zundert J., Patijn J., Kessels A., Lamé I., van Suijlekom H., van Kleef M. (2007). Pulsed Radiofrequency Adjacent to the Cervical Dorsal Root Ganglion in Chronic Cervical Radicular Pain: A Double Blind Sham Controlled Randomized Clinical Trial. Pain.

[26] Bharti N., Sujith J., Singla N., Panda N.B., Bala I. (2019). Radiofrequency Thermoablation of the Gasserian Ganglion versus the Peripheral Branches of the Trigeminal Nerve for Treatment of Trigeminal Neuralgia: A Randomized, Control Trial. Pain Physician.

[27] Masala S., Cuzzolino A., Morini M., Raguso M., Fiori R. (2018). Ultrasound-Guided Percutaneous Radiofrequency for the Treatment of Morton’s Neuroma. Cardiovasc. Interv. Radiol..

[28] Alférez M.D., Corda A., de Blas I., Gago L., Fernandes T., Rodríguez-Piza I., Balañá B., Corda F., Gómez Ochoa P. (2024). Percutaneous Ultrasound-Guided Radiofrequency Ablation as a Therapeutic Approach for the Management of Insulinomas and Associated Metastases in Dogs. Animals.

[29] Wright K.N., Connor C.E., Irvin H.M., Knilans T.K., Webber D., Kass P.H. (2018). Atrioventricular Accessory Pathways in 89 Dogs: Clinical Features and Outcome after Radiofrequency Catheter Ablation. J. Vet. Intern. Med..

[30] Choi E.J., Choi Y.M., Jang E.J., Kim J.Y., Kim T.K., Kim K.H. (2016). Neural Ablation and Regeneration in Pain Practice. Korean J. Pain.

[31] Chu K.F., Dupuy D.E. (2014). Thermal Ablation of Tumours: Biological Mechanisms and Advances in Therapy. Nat. Rev. Cancer.

[32] Dong Y., Chen Y., Yao B., Song P., Xu R., Li R., Liu P., Zhang Y., Mu L., Tong X. (2022). Neuropathologic Damage Induced by Radiofrequency Ablation at Different Temperatures. Clinics.

[33] Zachariah C., Mayeux J., Alas G., Adesina S., Mistretta O.C., Ward P.J., Chen A., English A.W., Washington A.V. (2020). Physiological and Functional Responses of Water-Cooled versus Traditional Radiofrequency Ablation of Peripheral Nerves in Rats. Reg. Anesth. Pain Med..

[34] Lee S.H., Kang C.H., Lee S.H., Derby R., Yang S.N., Lee J.E., Kim J.H., Kim S.S., Lee J.H. (2008). Ultrasound-Guided Radiofrequency Neurotomy in Cervical Spine: Sonoanatomic Study of a New Technique in Cadavers. Clin. Radiol..

[35] Amari M., Rabbogliatti V., Ravasio G., Auletta L., Brioschi F.A., Riccaboni P., Aere S.D., Roccabianca P. (2024). Development of an Ultrasound-Guided Radiofrequency Ablation Technique in the Equine Cadaveric Distal Limb: Histological Findings and Potential for Treating Chronic Lameness. Front. Vet. Sci..

[36] Boesch J.M., Campoy L., Southard T., Dewey C., Erb H.N., Gleed R.D., Martin-Flores M., Sakai D.M., Sutton J., Williamson B. (2019). Histological, Electrophysiological and Clinical Effects of Thermal Radiofrequency Therapy of the Saphenous Nerve and Pulsed Radiofrequency Therapy of the Sciatic Nerve in Dogs. Vet. Anaesth. Analg..

[37] Vatansever D., Tekin I., Tuglu I., Erbuyun K., Ok G. (2008). A Comparison of the Neuroablative Effects of Conventional and Pulsed Radiofrequency Techniques. Clin. J. Pain.

[38] Sam J., Catapano M., Sahni S., Ma F., Abd-Elsayed A., Visnjevac O. (2021). Pulsed Radiofrequency in Interventional Pain Management: Cellular and Molecular Mechanisms of Action—An Update and Review. Pain Physician.

[39] Ren H., Jin H., Jia Z., Ji N., Luo F. (2018). Pulsed Radiofrequency Applied to the Sciatic Nerve Improves Neuropathic Pain by Down-Regulating The Expression of Calcitonin Gene-Related Peptide in the Dorsal Root Ganglion. Int. J. Med. Sci..

[40] Erdine S., Bilir A., Cosman E.R., Cosman E.R. (2009). Ultrastructural Changes in Axons Following Exposure to Pulsed Radiofrequency Fields. Pain Pract..

[41] Shi Y., Wu W. (2016). Treatment of Neuropathic Pain Using Pulsed Radiofrequency: A Meta-Analysis. Pain Physician.

[42] Almuhanna A.H., Cahalan S.D., Lane A., Goodwin D., Perkins J., Piercy R.J. (2021). Optimisation and Validation of Immunohistochemical Axonal Markers for Morphological and Functional Characterisation of Equine Peripheral Nerves. Equine Vet. J..

[43] Bassage L.H., Ross M.W., Ross M.W., Dyson S.J. (2003). Diagnostic Analgesia. Diagnosis and Management of Lameness in the Horse.

[44] Baxter G.M., Baxter G.M. (2021). Examination for Lameness—Perineural and Intrasynovial Anesthesia. Adams and Stashak’s Lameness in Horses.

[45] Moyer W. (2011). Equine Joint Injection and Regional Anesthesia.

[46] Denoix J.M. (1994). Diagnostic Techniques for Identification and Documentation of Tendon and Ligament Injuries. Vet. Clin. N. Am. Equine Pract..

[47] Keegan K.G., Baxter G.M. (2021). Examination for Lameness—Subjective Assessment of Lameness. Adams and Stashak’s Lameness in Horses.

[48] Young J.M., Schoonover M.J., Kembel S.L., Taylor J.D., Bauck A.G., Gilliam L.L. (2020). Efficacy of Orally Administered Gabapentin in Horses with Chronic Thoracic Limb Lameness. Vet. Anaesth. Analg..

[49] Wray J.K., Dixon B., Przkora R., StatPearls Publishing (2023). Radiofrequency Ablation. StatPearls.

[50] Alvites R., Rita Caseiro A., Santos Pedrosa S., Vieira Branquinho M., Ronchi G., Geuna S., Varejão A.S.P., Colette Maurício A. (2018). Peripheral Nerve Injury and Axonotmesis: State of the Art and Recent Advances. Cogent Med..

[51] Konig H.E., Liebich H.-G., Konig H.E., Liebich H.-G. (2007). Nervous System (Systema Nervosum). Veterinary Anatomy of Domestic Animals.

[52] LeBlanc A.K., Atherton M., Bentley R.T., Boudreau C.E., Burton J.H., Curran K.M., Dow S., Giuffrida M.A., Kellihan H.B., Mason N.J. (2021). Veterinary Cooperative Oncology Group—Common Terminology Criteria for Adverse Events (VCOG-CTCAE v2) Following Investigational Therapy in Dogs and Cats. Vet. Comp. Oncol..

[53] Lu K., Liliang P.C., Liang C.L., Wang K.W., Tsai Y.D., Chen H.J. (2012). Efficacy of Conventional and Pulsed Radiofrequency for Treating Chronic Lumbar Facet Joint Pain. Formos. J. Surg..

[54] Agarwal A., Rastogi S., Bansal M., Kumar S., Malviya D., Thacker A.K. (2021). Radiofrequency Treatment of Idiopathic Trigeminal Neuralgia (Conventional vs. Pulsed): A Prospective Randomized Control Study. Anesth. Essays Res..

[55] Choi W.J., Hwang S.J., Song J.G., Leem J.G., Kang Y.U., Park P.H., Shin J.W. (2011). Radiofrequency Treatment Relieves Chronic Knee Osteoarthritis Pain: A Double-Blind Randomized Controlled Trial. Pain.

[56] Gazelka H.M., Knievel S., Mauck W.D., Moeschler S.M., Pingree M.J., Rho R.H., Lamer T.J. (2014). Incidence of Neuropathic Pain after Radiofrequency Denervation of the Third Occipital Nerve. J. Pain Res..

[57] Boesch J.M., Campoy L., Martin-Flores M., Gleed R.D. (2020). Thermal Radiofrequency Ablation of the Saphenous Nerve in Dogs with Pain from Naturally-Occurring Stifle Osteoarthritis. Vet. Anaesth. Analg..

[58] Cosman E.R., Dolensky J.R., Hoffman R.A. (2014). Factors That Affect Radiofrequency Heat Lesion Size. Pain Med..

[59] Künzle A., van Kuijk S.M.J., Koetsier E. (2023). Efficacy of Cervical Facet Joint Radiofrequency Ablation Using a Multitined Cannula, a Technical Note, and Observational Study. Pain Physician.

[60] Kidd V.D., Strum S.R., Strum D.S., Shah J. (2019). Genicular Nerve Radiofrequency Ablation for Painful Knee Arthritis: The Why and the How. JBJS Essent. Surg. Tech..

[61] Provenzano D.A., Lassila H.C., Somers D. (2010). The Effect of Fluid Injection on Lesion Size during Radiofrequency Treatment. Reg. Anesth. Pain Med..

[62] Sheiman R.G., Mullan C., Ahmed M. (2012). In Vivo Determination of a Modified Heat Capacity of Small Hepatocellular Carcinomas Prior to Radiofrequency Ablation: Correlation with Adjacent Vasculature and Tumour Recurrence. Int. J. Hyperth..

[63] Carpenedo R., Al-Wardat M., Vizzolo L., Germani G., Chinè E., Ridolfo S., Dauri M., Natoli S. (2022). Ultrasound-Guided Pulsed Radiofrequency of the Saphenous Nerve for Knee Osteoarthritis Pain: A Pilot Randomized Trial. Pain Manag..

[64] Fang L., Tao W., Jingjing L., Nan J. (2015). Comparison of High-Voltage- with Standard-Voltage Pulsed Radiofrequency of Gasserian Ganglion in the Treatment of Idiopathic Trigeminal Neuralgia. Pain Pract..

[65] Jia Y., Cheng H., Shrestha N., Ren H., Zhao C., Feng K., Luo F. (2023). Effectiveness and Safety of High-Voltage Pulsed Radiofrequency to Treat Patients with Primary Trigeminal Neuralgia: A Multicenter, Randomized, Double-Blind, Controlled Study. J. Headache Pain.

[66] Choi S., Choi H.J., Cheong Y., Lim Y.J., Park H.K. (2013). Internal-Specific Morphological Analysis of Sciatic Nerve Fibers in a Radiofrequency-Induced Animal Neuropathic Pain Model. PLoS ONE.

[67] Bowker R.M., Linder K., Sonea I.M., Guida L.A. (1995). Sensory Nerve Fibres and Receptors in Equine Distal Forelimbs and Their Potential Roles in Locomotion. Equine Vet. J..

[68] Facchini G., Spinnato P., Guglielmi G., Albisinni U., Bazzocchi A. (2017). A Comprehensive Review of Pulsed Radiofrequency in the Treatment of Pain Associated with Different Spinal Conditions. Br. J. Radiol..

[69] Roca G., de Andrés Ares J., Luisa Franco Gay M., Nieto C., Teresa Bovaira M. (2014). Radiofrequency Techniques: Complications and Troubleshooting. Tech. Reg. Anesth. Pain Manag..

[70] Tzaan W.C., Tasker R.R. (2000). Percutaneous Radiofrequency Facet Rhizotomy—Experience with 118 Procedures and Reappraisal of Its Value. Can. J. Neurol. Sci..

[71] Rodríguez-Merchán E.C., Delgado-Martínez A.D., De Andrés-Ares J. (2023). Upper Limb and Lower Limb Radiofrequency Treatments in Orthopaedics. EFORT Open Rev..

[72] Reddy A.T., Goyal N., Cascio M., Leal J., Singh K. (2023). Abnormal Paresthesias Associated with Radiofrequency Ablation of Lumbar Medial Branch Nerves: A Case Report. Cureus.

[73] Boswell M.V., Colson J.D., Sehgal N., Dunbar E.E., Epter R. (2007). A Systematic Review of Therapeutic Facet Joint Interventions in Chronic Spinal Pain. Pain Physician.

[74] Stolzenberg D., Gordin V., Vorobeychik Y. (2014). Incidence of Neuropathic Pain after Cooled Radiofrequency Ablation of Sacral Lateral Branch Nerves. Pain Med..

[75] Kornick C., Kramarich S.S., Lamer T.J., Sitzman B.T. (2004). Complications of Lumbar Facet Radiofrequency Denervation. Spine.

[76] Erken H.Y., Ayanoglu S., Akmaz I., Erler K., Kiral A. (2014). Prospective Study of Percutaneous Radiofrequency Nerve Ablation for Chronic Plantar Fasciitis. Foot Ankle Int..

[77] Huang Y.H., Hou S.Y., Cheng J.K., Wu C.H., Lin C.R. (2016). Pulsed Radiofrequency Attenuates Diabetic Neuropathic Pain and Suppresses Formalin-Evoked Spinal Glutamate Release in Rats. Int. J. Med. Sci..

[78] Tanaka N., Yamaga M., Tateyama S., Uno T., Tsuneyoshi I., Takasaki M. (2010). The Effect of Pulsed Radiofrequency Current on Mechanical Allodynia Induced with Resiniferatoxin in Rats. Anesth. Analg..

[79] Hsu M. (2014). Significance of Clinical Treatments on Peripheral Nerve and Its Effect on Nerve Regeneration. J. Neurol. Disord..

[80] Schmidt S., Karri J., Singh M., Abd-Elsayed A. (2021). Incidence, Diagnosis, and Management of Neuromas Following Radiofrequency Ablation Treatment: A Narrative Review. Curr. Pain Headache Rep..

[81] Gekht G., Nottmeier E.W., Lamer T.J. (2010). Painful Medial Branch Neuroma Treated with Minimally Invasive Medial Branch Neurectomy. Pain Med..

[82] Matthews S., Dart A.J., Dowling B.A. (2003). Palmar Digital Neurectomy in 24 Horses Using the Guillotine Technique. Aust. Vet. J..

[83] Jackman B.R., Baxter G.M., Doran R.E., Allen D., Parks A.H. (1993). Palmar Digital Neurectomy in Horses 57 Cases (1984–1990). Vet. Surg..

[84] Dabareiner R.M., White N.A., Sullins K.E. Comparison of Current Techniques for Palmar Digital Neurectomy in Horses. Proceedings of the Annual Convention of the AAEP 1997.

[85] Maher O., Davis D.M., Drake C., Myhre G.D., Labbe K.M., Han J.H., Lejeune S.S. (2008). Pull-through Technique for Palmar Digital Neurectomy: Forty-One Horses (1998–2004). Vet. Surg..

[86] Belba A., Vanneste T., Jerjir A., Smeets K., Van Buyten J.P., Bellemans J., Van Zundert J. (2022). Complex Regional Pain Syndrome of the Knee after Conventional Radiofrequency Ablation of the Genicular Nerves Treated Successfully with Dorsal Root Ganglion Stimulation: A Case Report. Pain Pract..

[87] Guerra J.A., Schumacher J., Salcedo-Jiménez R., Rohrbach B.W., Monterde A.R., Romero L.R., Donnell R. (2022). Denervating the Pelvic Suspensory Ligaments of Horses Causes Morphological and Histological Changes in the Ligaments. Am. J. Vet. Res..

[88] Eggleston R.B., Baxter G.M., Baxter G.M. (2021). Lameness of the Distal Limb—Navicular Region/Palmar Foot. Adams and Stashak’s Lameness in Horses.

[89] Cohen S.P., Mao J. (2014). Neuropathic Pain: Mechanisms and Their Clinical Implications. BMJ.

[90] McDonnell S.M. (2008). Practical Review of Self-Mutilation in Horses. Anim. Reprod. Sci..

[91] Zipu J., Hao R., Chunmei Z., Lan M., Ying S., Fang L. (2021). Long-Term Follow-up of Pulsed Radiofrequency Treatment for Trigeminal Neuralgia: Kaplan-Meier Analysis in a Consecutive Series of 149 Patients. Pain Physician.

[92] Byrd D., Mackey S. (2008). Pulsed Radiofrequency for Chronic Pain. Curr. Pain Headache Rep..

[93] Malaithong W., Munjupong S. (2022). Combined Continuous Radiofrequency Ablation and Pulsed Neuromodulation to Treat Cervical Facet Joint Pain and Alleviate Postcervical Radiofrequency Side Effects. Anesth. Pain Med..

[94] Zhao W.X., Wang Q., He M.W., Yang L.Q., Wu B.S., Ni J.X. (2015). Radiofrequency Thermocoagulation Combined with Pulsed Radiofrequency Helps Relieve Postoperative Complications of Trigeminal Neuralgia. Genet. Mol. Res..

[95] Alexander K., Dobson H. (2003). Ultrasonography of Peripheral Nerves in the Normal Adult Horse. Vet. Radiol. Ultrasound.

