# Supplementary material for: Ultrasound-Guided Radiofrequency Ablation and Pulsed Radiofrequency Treatment for Chronic Lameness Due to Distal Forelimb Disease in Horses: A Pilot Study
Source: Animals (Basel). 2025 Aug 10;15(16):2341. doi: 10.3390/ani15162341 (PMC12383019; doi:10.3390/ani15162341)
Supplement: Supplementary file 1 [file animals-15-02341-s001.zip › Table S1.pdf]

Table S1. Signalment data for the horses included in the radiofrequency ablation group (N= 8) and in the pulsed radiofrequency group (N= 16), including specific temperature and minute setting received, treated limb(s), breed, sex, weight, age, work type, pathological category, and specific pathology.

Case	Group	Setting	Forelimb	Breed	Sex	Weight (Kg)	Age (Years)	Work type	Pathological Category	Pathology
**1**	RFA	VERY HIGH	R	Dutch Warmblood	M	495	12	Jumping	tenodesmic	Proximal digital annular ligament desmopathy
**2**	RFA	VERY HIGH	R	Pure Spanish horse	G	480	12	Dressage	tenodesmic	DDFT tendinopathy + Collateral ligament of DIPJ desmopathy
**3**	RFA	HIGH	BILAT	Pure Spanish horse	M	571	11	Dressage	tenodesmic	DDFT tendinopathy
**4**	RFA	MEDIUM	L	Criollo	G	427	7	Flatwork	Joint	PIPJ OA
**5**	RFA	MEDIUM	R	Quarter horse	F	447	9	Western	Osseous	Navicular syndrome + DDFT tendinopathy + DSL desmopathy
**6**	RFA	LOW	L	Hannover	F	564	10	Jumping	Osseous	Navicular syndrome
**7**	RFA	LOW	L	Dutch Warmblood	G	489	4	Dressage	Osseous	Chronic navicular bone fracture
**8**	RFA	LOW	R	Dutch Warmblood	M	500	17	Jumping	Joint	DIPJ OA
**1**	PRF	42 °C, 12 min	R	Quarter horse	F	460	8	Western	tenodesmic	DDFT tendinopathy + collateral ligament of navicular bone desmopathy
**2**	PRF	42 °C, 12 min	BILAT	American paint horse	F	523	25	Western	Joint	DIPJ and PIPJ OA
**3**	PRF	42 °C, 12 min	R	Holsteiner	F	611	14	Jumping	tenodesmic	DDFT tendinopathy + collateral ligament of DIPJ desmopathy
**4**	PRF	42 °C, 12 min	BILAT	Holsteiner	G	668	19	Flatwork	Joint	DIPJ OA
**5**	PRF	42 °C, 12 min	BILAT	Quarter horse	F	451	3	Western	Osseous	Navicular syndrome + DDFT tendinopathy
**6**	PRF	42 °C, 12 min	R	Quarter horse	G	532	10	Western	Osseous	Navicolar syndrome
**7**	PRF	42 °C, 12 min	BILAT	Quarter horse	F	444	10	Western	Osseous	Navicolar syndrome

**8**	PRF	42 °C, 12 min	BILAT	Pure Spanish horse	G	504	24	Paddock	Joint	DIPJ and PIPJ OA + navicolar syndrome
**9**	PRF	42 °C, 12 min	L	Quarter horse	G	472	15	Western	tenodesmic	DDFT tendinopathy
**10**	PRF	42 °C, 12 min	L	Hannover	G	490	13	Dressage	Joint	DIPJ OA + Navicular syndrome
**11**	PRF	42 °C, 12 min	BILAT	Hannover	F	511	20	Flatwork	Joint	PIPJ OA + Navicolar syndrome
**12**	PRF	42 °C, 12 min	BILAT	Haflinger	F	446	12	Trekking	Joint	PIPJ OA
**13**	PRF	42 °C, 12 min	R	Quarter horse	G	433	28	Flatwork	Joint	DIPJ and PIPJ OA
**14**	PRF	42 °C, 12 min	BILAT	Dutch Warmblood	G	620	16	Paddock	tenodesmic	DSL desmopathy + collateral ligament of navicular bone desmopathy
**15**	PRF	42 °C, 12 min	L	Zangersheide	F	489	7	Jumping	tenodesmic	DDFT tendinopathy + DSL desmopathy
**16**	PRF	42 °C, 12 min	R	Oldenbourg	G	540	18	Jumping	Joint	DIPJ OA

BILAT: Bilateral treatment; DDFT: Deep Digital Flexor Tendon; DIPJ: Distal Interphalangeal Joint; DSL: Distal Sesamoidean Ligament; F: Female; G: Gelding; HIGH: 90 °C for 2 minutes; L: Left forelimb; LOW: 60 °C for 6 minutes; M: Intact male; MEDIUM: 70 °C for 4 minutes; OA: Osteoarthritis; PIPJ: Proximal Interphalangeal Joint; PRF: Pulsed Radiofrequency; R: Right forelimb; RFA: Radiofrequency Ablation; VERY HIGH: 80 °C for 8 minutes.
