# Supplementary material for: Ultrasound-Guided Radiofrequency Ablation and Pulsed Radiofrequency Treatment for Chronic Lameness Due to Distal Forelimb Disease in Horses: A Pilot Study
Source: Animals (Basel). 2025 Aug 10;15(16):2341. doi: 10.3390/ani15162341 (PMC12383019; doi:10.3390/ani15162341)
Supplement: Supplementary file 1 [file animals-15-02341-s001.zip › Table S2.pdf]

Table S2. Summary of clinical outcomes and lameness scores for horses over the entire follow-up period. Individual horse data including treatment group, specific temperature and minute setting received, outcome after the first and (if applicable) second treatment, complications, return to previous work activity, and lameness scores at baseline and at each follow-up.

Case	Group	Setting	Outcome 1° treat	Complications	Received second treatment?	Outcome 2° treat	Return to previous level of work	AAEP T0	AAEP T1	AAEP T2	AAEP T3	AAEP T4	AAEP T5	AAEP T6
**1**	RFA	VERY HIGH	Failed	Allodynia	YES	Successful	YES	3	3	3	1	0	0	0
**2**	RFA	VERY HIGH	Failed	Allodynia	YES	Successful	YES	3	3	4	1	1	1	1
**3**	RFA	HIGH	Failed	Pain/Lameness Increased	YES	Successful	YES	2	4	3	1	0	0	0
**4**	RFA	MEDIUM	Failed	Soft tissue wound pastern region	Excluded			3	1	4				
**5**	RFA	MEDIUM	Partial	NO	YES	Successful	YES	3	2	2	1	0	0	0
**6**	RFA	LOW	Failed	Localized oedema	YES	Partial	YES	2	2	2	1	1	1	1
**7**	RFA	LOW	Partial	NO	YES	Successful	YES	3	3	2	0	0	0	0
**8**	RFA	LOW	Failed	Allodynia	YES	Successful	YES	3	3	3	1	0	0	0
**1**	PRF	42 °C, 12 min	Partial	NO	YES	Successful	NO	2	2	1	0	0	0	1
**2**	PRF	42 °C, 12 min	Failed	NO	YES	Successful	YES	3	3	3	1	0	0	0
**3**	PRF	42 °C, 12 min	Successful	NO	NO		YES	2	0	0	0	0	0	0
**4**	PRF	42 °C, 12 min	Successful	NO	NO		YES	2	0	0	0	0	0	0
**5**	PRF	42 °C, 12 min	Successful	NO	YES	Successful	YES	3	2	1	0	0	0	0
**6**	PRF	42 °C, 12 min	Failed	NO	YES	Successful	YES	3	3	3	1	0	0	0

**7**	PRF	42 °C, 12 min	Successful	NO	YES	Successful	NO	4	3	2	1	0	0	1
**8**	PRF	42 °C, 12 min	Partial	NO	YES	Partial	YES	4	3	3	2	2	2	2
**9**	PRF	42 °C, 12 min	Partial	NO	YES	Successful	YES	3	2	2	0	0	0	1
**10**	PRF	42 °C, 12 min	Partial	Pain/Lameness increased	YES	Failed	NO	2	3	1	1	1	1	2
**11**	PRF	42 °C, 12 min	Successful	NO	NO		YES	2	0	0	0	0	0	0
**12**	PRF	42 °C, 12 min	Successful	NO	NO		YES	4	3	2	2	1	1	1
**13**	PRF	42 °C, 12 min	Failed	NO	YES	Failed	NO	3	3	3	3	3	3	3
**14**	PRF	42 °C, 12 min	Successful	NO	NO		YES	3	2	0	0	0	0	0
**15**	PRF	42 °C, 12 min	Successful	NO	NO		YES	2	1	0	0	0	0	0
**16**	PRF	42 °C, 12 min	Successful	NO	NO		YES	3	2	1	1	1	1	1

AAEP: American Association of Equine Practitioners lameness scale (score 0–5); HIGH: 90 °C for 2 minutes; LOW: 60 °C for 6 minutes; MEDIUM: 70 °C for 4 minutes; PRF: Pulsed Radiofrequency; RFA: Radiofrequency Ablation; VERY HIGH: 80 °C for 8 minutes.
